# *catena*-Poly[triethyl­ammonium [[triphenyl­tin(IV)]-μ-3,3′-dihydroxy-4,4′-methyl­ene­di-2-naphtho­ato]]

**DOI:** 10.1107/S1600536810052402

**Published:** 2010-12-24

**Authors:** Lijun Liu, Shuwen Gong

**Affiliations:** a College of Chemistry and Chemical Engineering, Liaocheng University, Shandong 252059, People’s Republic of China; b Department of Chemistry, Liaocheng University, Liaocheng 252059, People’s Republic of China

## Abstract

The title compound, {(C_6_H_16_N)[Sn(C_6_H_5_)_3_(C_23_H_14_O_6_)]}_*n*_, has an infinite chain structure, formed through monodentate carboxyl­ate groups of the pamoic acid anion. The anion bridges two symmetry-related Sn(IV) ions and the resulting polymeric chains are parallel to [201] in the crystal. Et_3_NH^+^ cations are inserted between the chains. The coordination of the Sn(IV) atom is completed by three phenyl ligands, giving a distorted trigonal–bipyramidal geometry.

## Related literature

For related polymeric organotin structures, see: Ma *et al.* (2008 ▶).
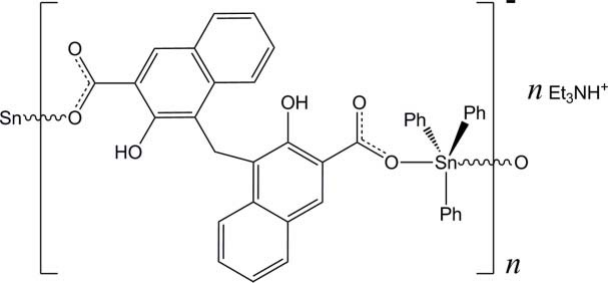


## Experimental

### 

#### Crystal data

(C_6_H_16_N)[Sn(C_6_H_5_)_3_(C_23_H_14_O_6_)]
*M* *_r_* = 838.53
Monoclinic, 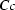

*a* = 13.2590 (14) Å
*b* = 16.3231 (16) Å
*c* = 19.166 (2) Å
β = 98.580 (2)°
*V* = 4101.6 (7) Å^3^
*Z* = 4
Mo *K*α radiation
μ = 0.67 mm^−1^
*T* = 298 K
0.24 × 0.14 × 0.11 mm

#### Data collection

Siemens SMART CCD 1000 area-detector diffractometer
Absorption correction: multi-scan (*SADABS*; Siemens, 1996 ▶) *T*_min_ = 0.855, *T*_max_ = 0.930
10588 measured reflections
6019 independent reflections
4406 reflections with *I* > 2σ(*I*)
*R*_int_ = 0.115

#### Refinement

*R*[*F* ^2^ > 2σ(*F* ^2^)] = 0.070
*wR*(*F* ^2^) = 0.182
*S* = 0.98
6019 reflections
499 parameters
2 restraints
H-atom parameters constrained
Δρ_max_ = 1.53 e Å^−3^
Δρ_min_ = −0.84 e Å^−3^
Absolute structure: Flack (1983 ▶), 2394 Friedel pairs
Flack parameter: −0.04 (4)

### 

Data collection: *SMART* (Siemens, 1996 ▶); cell refinement: *SAINT* (Siemens, 1996 ▶); data reduction: *SAINT*; program(s) used to solve structure: *SHELXS97* (Sheldrick, 2008 ▶); program(s) used to refine structure: *SHELXL97* (Sheldrick, 2008 ▶); molecular graphics: *SHELXTL* (Sheldrick, 2008 ▶); software used to prepare material for publication: *SHELXTL*.

## Supplementary Material

Crystal structure: contains datablocks I, global. DOI: 10.1107/S1600536810052402/bh2324sup1.cif

Structure factors: contains datablocks I. DOI: 10.1107/S1600536810052402/bh2324Isup2.hkl

Additional supplementary materials:  crystallographic information; 3D view; checkCIF report

## Figures and Tables

**Table:** 

Sn1—C36	2.154 (15)
Sn1—C24	2.157 (14)
Sn1—C30	2.180 (11)
Sn1—O4	2.227 (12)
Sn1—O1	2.314 (12)

**Table:** 

C36—Sn1—C24	110.7 (4)
C36—Sn1—C30	139.3 (5)
C24—Sn1—C30	109.5 (5)
C36—Sn1—O4	91.1 (5)
C24—Sn1—O4	89.3 (5)
C30—Sn1—O4	95.1 (4)
C36—Sn1—O1	86.3 (5)
C24—Sn1—O1	89.2 (5)
C30—Sn1—O1	88.6 (4)
O4—Sn1—O1	176.3 (4)

**Fig. 1.**
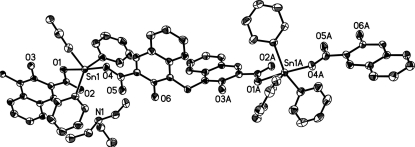
The molecular structure of the compound, showing 30% probability displacement ellipsoids.

**Fig. 2.**
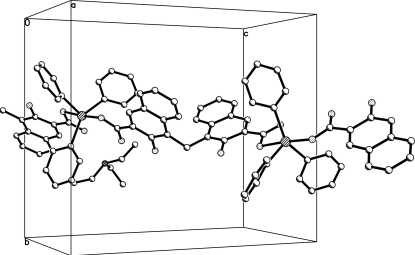
The unit cell of the title compound.

## supplementary crystallographic information

### Comment

The title compound is a one-dimensional infinite chain structure linked by nomodentate pamoic acid ligand, as shown in Figures 1 and 2. The geometry of tin atoms is distorted trigonal bipyramidal, surrounded axially by two oxygen atoms and equatorially by three carbon atoms of the phenyl groups. The axial angle O4—Sn1—O1 is 176.3 (4)°, close to linear arrangement. Three Sn-phenyl groups define the equatorial plane and the sum of the trigonal C—Sn—C angles is 359.5°, as expected for a bipyramidal geometry. The Sn1—O1 distance is 2.314 (12) Å, which a bit longer than the covalent bond length Sn—O, but similar to those found in other reported triorganotin polymeric structures (*e.g*. Ma *et al.*, 2008).

### Experimental

The reaction was carried out under nitrogen atmosphere. 4,4'-Methylenebis(3-hydroxy-2-naphthoic acid) (1 mmol) and triethylamine (2 mmol) were added to a stirred solution of benzene (30 ml) in a Schlenk flask and stirred for 0.5 h. Triphenyltin chloride (2 mmol) was then added and the reaction mixture was stirred for 12 h at 353 K. The resulting clear solution was evaporated under vacuum. The product was crystallized from dichloromethane to yield colourless blocks of the title complex (yield 83%. m.p. 458 K). Anal. Calcd (%) for C_47_H_45_N_1_O_6_Sn_1_ (Mr = 838.53): C 67.32, H 5.41. Found (%): C 67.61, H, 5.68.

### Refinement

All H atoms were placed geometrically and treated as riding on their parent atoms with C—H = 0.93 (aromatic CH), C—H = 0.97 (methylene), C—H = 0.96 (methyl), N—H = 0.91, and O—H = 0.82 Å. Isotropic displacement parameters for H atoms were calculated as *U*_iso_(H) = 1.2*U*_eq_(carrier atom) or *U*_iso_(H) = 1.5*U*_eq_(carrier atom).

### Crystal data

**Table:**

(C_6_H_16_N)[Sn(C_6_H_5_)_3_(C_23_H_14_O_6_)]	*F*(000) = 1728
*M**_r_* = 838.53	*D*_x_ = 1.358 Mg m^−^^3^
Monoclinic, *C**c*	Melting point: 458 K
Hall symbol: C -2yc	Mo *K*α radiation, λ = 0.71073 Å
*a* = 13.2590 (14) Å	Cell parameters from 2599 reflections
*b* = 16.3231 (16) Å	θ = 2.4–25.0°
*c* = 19.166 (2) Å	µ = 0.67 mm^−^^1^
β = 98.580 (2)°	*T* = 298 K
*V* = 4101.6 (7) Å^3^	Block, colourless
*Z* = 4	0.24 × 0.14 × 0.11 mm

### Data collection

**Table:**

Bruker SMART CCD 1000 area-detector diffractometer	6019 independent reflections
Radiation source: fine-focus sealed tube	4406 reflections with *I* > 2σ(*I*)
graphite	*R*_int_ = 0.115
φ and ω scans	θ_max_ = 25.0°, θ_min_ = 2.0°
Absorption correction: multi-scan (*SADABS*; Siemens, 1996)	*h* = −15→13
*T*_min_ = 0.855, *T*_max_ = 0.930	*k* = −15→19
10588 measured reflections	*l* = −22→22

### Refinement

**Table:**

Refinement on *F*^2^	Secondary atom site location: difference Fourier map
Least-squares matrix: full	Hydrogen site location: inferred from neighbouring sites
*R*[*F*^2^ > 2σ(*F*^2^)] = 0.070	H-atom parameters constrained
*wR*(*F*^2^) = 0.182	*w* = 1/[σ^2^(*F*_o_^2^) + (0.1004*P*)^2^] where *P* = (*F*_o_^2^ + 2*F*_c_^2^)/3
*S* = 0.98	(Δ/σ)_max_ = 0.001
6019 reflections	Δρ_max_ = 1.53 e Å^−^^3^
499 parameters	Δρ_min_ = −0.84 e Å^−^^3^
2 restraints	Absolute structure: Flack (1983), 2394 Friedel pairs
0 constraints	Flack parameter: −0.04 (4)
Primary atom site location: structure-invariant direct methods	

### Fractional atomic coordinates and isotropic or equivalent isotropic displacement parameters (Å^2^)

**Table:**

	*x*	*y*	*z*	*U*_iso_*/*U*_eq_	
Sn1	0.76424 (5)	0.44960 (4)	0.08738 (4)	0.0419 (2)	
N1	0.5704 (7)	0.6521 (6)	0.2187 (5)	0.059 (2)	
H1	0.5506	0.6055	0.1943	0.071*	
O1	0.5920 (9)	0.4307 (6)	0.0488 (6)	0.047 (2)	
O2	0.5631 (6)	0.5121 (5)	0.1380 (4)	0.0522 (18)	
O3	0.4525 (6)	0.4040 (5)	−0.0559 (4)	0.055 (2)	
H3	0.5079	0.4003	−0.0305	0.082*	
O4	0.9306 (9)	0.4593 (6)	0.1263 (6)	0.053 (3)	
O5	0.9078 (7)	0.5519 (5)	0.2087 (5)	0.062 (2)	
O6	1.0543 (6)	0.5914 (5)	0.3030 (4)	0.057 (2)	
H6	0.9977	0.5932	0.2790	0.086*	
C1	0.5324 (9)	0.4761 (7)	0.0813 (6)	0.046 (3)	
C2	0.3574 (9)	0.5325 (6)	0.0775 (6)	0.046 (3)	
H2	0.3789	0.5536	0.1223	0.055*	
C3	0.4245 (8)	0.4854 (7)	0.0451 (6)	0.046 (3)	
C4	0.3884 (14)	0.4520 (7)	−0.0235 (9)	0.044 (4)	
C5	0.2893 (10)	0.4648 (8)	−0.0581 (7)	0.043 (3)	
C6	0.2219 (8)	0.5155 (7)	−0.0229 (6)	0.045 (2)	
C7	0.2572 (8)	0.5490 (6)	0.0438 (6)	0.043 (2)	
C8	0.1909 (9)	0.6002 (7)	0.0782 (6)	0.054 (3)	
H8	0.2134	0.6211	0.1230	0.065*	
C9	0.0950 (9)	0.6178 (8)	0.0452 (6)	0.056 (3)	
H9	0.0535	0.6536	0.0658	0.067*	
C10	0.0601 (9)	0.5819 (8)	−0.0195 (7)	0.058 (3)	
H10	−0.0069	0.5915	−0.0402	0.069*	
C11	0.1191 (9)	0.5338 (7)	−0.0532 (6)	0.051 (3)	
H11	0.0927	0.5120	−0.0969	0.061*	
C12	0.9632 (9)	0.5017 (8)	0.1802 (6)	0.051 (3)	
C13	1.1374 (14)	0.4399 (9)	0.1843 (10)	0.047 (4)	
H13	1.1113	0.4120	0.1433	0.056*	
C14	1.0735 (8)	0.4935 (7)	0.2120 (6)	0.045 (2)	
C15	1.1138 (8)	0.5372 (6)	0.2741 (5)	0.041 (2)	
C16	1.2141 (8)	0.5256 (6)	0.3074 (6)	0.043 (2)	
C17	1.2795 (9)	0.4682 (7)	0.2773 (7)	0.043 (3)	
C18	1.2383 (10)	0.4244 (8)	0.2135 (7)	0.048 (3)	
C19	1.3028 (8)	0.3696 (7)	0.1845 (6)	0.053 (3)	
H19	1.2765	0.3398	0.1445	0.063*	
C20	1.4025 (9)	0.3586 (8)	0.2130 (6)	0.058 (3)	
H20	1.4439	0.3232	0.1920	0.069*	
C21	1.4406 (10)	0.4009 (7)	0.2732 (7)	0.058 (3)	
H21	1.5079	0.3922	0.2935	0.070*	
C22	1.3820 (9)	0.4556 (7)	0.3046 (6)	0.049 (3)	
H22	1.4111	0.4845	0.3444	0.058*	
C23	0.2537 (9)	0.4235 (8)	−0.1279 (6)	0.045 (3)	
H23A	0.2001	0.3855	−0.1205	0.055*	
H23B	0.3102	0.3910	−0.1394	0.055*	
C24	0.7979 (12)	0.3698 (9)	0.0041 (8)	0.046 (3)	
C25	0.7434 (11)	0.2978 (8)	−0.0158 (6)	0.062 (3)	
H25	0.6855	0.2851	0.0041	0.075*	
C26	0.7759 (12)	0.2445 (8)	−0.0659 (7)	0.068 (4)	
H26	0.7404	0.1961	−0.0778	0.082*	
C27	0.8593 (13)	0.2632 (10)	−0.0969 (9)	0.070 (4)	
H27	0.8778	0.2291	−0.1317	0.084*	
C28	0.9164 (11)	0.3327 (9)	−0.0770 (6)	0.068 (4)	
H28	0.9743	0.3443	−0.0973	0.082*	
C29	0.8866 (9)	0.3854 (8)	−0.0263 (6)	0.059 (3)	
H29	0.9259	0.4314	−0.0124	0.071*	
C30	0.7482 (9)	0.5751 (7)	0.0484 (6)	0.049 (3)	
C31	0.6617 (11)	0.5948 (9)	−0.0028 (7)	0.069 (4)	
H31	0.6134	0.5553	−0.0190	0.082*	
C32	0.6520 (12)	0.6747 (9)	−0.0276 (7)	0.078 (4)	
H32	0.5945	0.6888	−0.0593	0.093*	
C33	0.7264 (12)	0.7352 (9)	−0.0065 (7)	0.075 (4)	
H33	0.7178	0.7888	−0.0226	0.090*	
C34	0.8115 (12)	0.7121 (8)	0.0385 (7)	0.072 (4)	
H34	0.8659	0.7483	0.0491	0.086*	
C35	0.8164 (11)	0.6348 (8)	0.0680 (6)	0.069 (4)	
H35	0.8701	0.6233	0.1037	0.083*	
C36	0.7411 (13)	0.3798 (10)	0.1791 (8)	0.054 (4)	
C37	0.7771 (11)	0.4021 (10)	0.2489 (6)	0.071 (4)	
H37	0.8141	0.4503	0.2579	0.086*	
C38	0.7585 (12)	0.3532 (10)	0.3055 (7)	0.079 (4)	
H38	0.7840	0.3682	0.3516	0.095*	
C39	0.7018 (14)	0.2820 (11)	0.2924 (9)	0.074 (5)	
H39	0.6892	0.2494	0.3299	0.088*	
C40	0.6642 (13)	0.2591 (9)	0.2248 (8)	0.078 (4)	
H40	0.6236	0.2126	0.2163	0.094*	
C41	0.6876 (11)	0.3068 (9)	0.1687 (6)	0.066 (3)	
H41	0.6665	0.2889	0.1228	0.079*	
C42	0.5879 (11)	0.7165 (9)	0.1637 (7)	0.072 (4)	
H42A	0.6405	0.6972	0.1376	0.086*	
H42B	0.6122	0.7666	0.1877	0.086*	
C43	0.4911 (13)	0.7356 (10)	0.1115 (8)	0.091 (5)	
H43A	0.4565	0.6854	0.0968	0.136*	
H43B	0.5094	0.7634	0.0711	0.136*	
H43C	0.4467	0.7699	0.1340	0.136*	
C44	0.4862 (11)	0.6733 (9)	0.2603 (7)	0.073 (4)	
H44A	0.4241	0.6826	0.2276	0.088*	
H44B	0.4745	0.6266	0.2894	0.088*	
C45	0.5061 (15)	0.7465 (11)	0.3066 (8)	0.104 (6)	
H45A	0.5742	0.7437	0.3319	0.156*	
H45B	0.4583	0.7477	0.3395	0.156*	
H45C	0.4987	0.7952	0.2782	0.156*	
C46	0.6704 (11)	0.6326 (9)	0.2641 (7)	0.073 (4)	
H46A	0.7178	0.6124	0.2342	0.087*	
H46B	0.6985	0.6828	0.2862	0.087*	
C47	0.6621 (14)	0.5712 (10)	0.3200 (8)	0.091 (5)	
H47A	0.6168	0.5912	0.3508	0.137*	
H47B	0.7283	0.5617	0.3467	0.137*	
H47C	0.6359	0.5208	0.2987	0.137*	

### Atomic displacement parameters (Å^2^)

**Table:**

	*U*^11^	*U*^22^	*U*^33^	*U*^12^	*U*^13^	*U*^23^
Sn1	0.0373 (3)	0.0414 (3)	0.0450 (3)	0.0002 (5)	−0.0006 (2)	−0.0007 (5)
N1	0.062 (6)	0.053 (6)	0.061 (6)	0.003 (5)	0.003 (5)	−0.009 (5)
O1	0.041 (6)	0.043 (5)	0.057 (6)	0.004 (4)	0.005 (4)	0.001 (4)
O2	0.048 (4)	0.055 (5)	0.050 (4)	0.006 (4)	−0.004 (3)	−0.006 (4)
O3	0.039 (4)	0.058 (5)	0.064 (5)	0.012 (4)	−0.004 (3)	−0.011 (4)
O4	0.042 (6)	0.063 (6)	0.050 (6)	−0.003 (5)	−0.005 (4)	−0.008 (5)
O5	0.043 (5)	0.072 (6)	0.067 (5)	0.008 (4)	−0.006 (4)	−0.010 (5)
O6	0.048 (5)	0.061 (5)	0.059 (5)	0.015 (4)	−0.003 (4)	−0.008 (4)
C1	0.046 (7)	0.044 (6)	0.047 (6)	0.001 (5)	0.004 (5)	0.007 (5)
C2	0.044 (7)	0.045 (6)	0.049 (6)	−0.004 (5)	0.008 (5)	−0.004 (5)
C3	0.037 (6)	0.046 (6)	0.053 (6)	−0.003 (5)	0.003 (5)	0.004 (5)
C4	0.038 (9)	0.039 (8)	0.052 (8)	0.007 (5)	0.003 (6)	0.000 (5)
C5	0.038 (7)	0.041 (7)	0.051 (7)	−0.004 (5)	0.007 (6)	0.001 (6)
C6	0.037 (6)	0.042 (6)	0.056 (6)	0.004 (5)	0.008 (5)	0.002 (5)
C7	0.035 (6)	0.039 (6)	0.054 (6)	−0.001 (5)	0.006 (4)	−0.001 (5)
C8	0.052 (7)	0.048 (7)	0.063 (7)	−0.003 (6)	0.006 (6)	−0.006 (6)
C9	0.037 (6)	0.056 (7)	0.076 (8)	0.003 (5)	0.012 (6)	0.001 (6)
C10	0.039 (7)	0.060 (7)	0.073 (8)	0.001 (6)	0.005 (6)	0.007 (6)
C11	0.039 (7)	0.051 (7)	0.061 (7)	0.004 (5)	0.002 (5)	0.003 (5)
C12	0.046 (7)	0.056 (8)	0.050 (6)	0.004 (6)	0.001 (5)	0.006 (6)
C13	0.036 (9)	0.049 (8)	0.054 (9)	−0.001 (6)	0.004 (6)	−0.004 (6)
C14	0.038 (6)	0.041 (6)	0.053 (6)	−0.002 (5)	0.002 (5)	0.000 (5)
C15	0.039 (6)	0.039 (6)	0.043 (5)	0.002 (5)	−0.002 (4)	0.001 (4)
C16	0.037 (6)	0.038 (6)	0.053 (6)	−0.002 (5)	0.000 (5)	0.002 (5)
C17	0.038 (7)	0.043 (7)	0.048 (7)	0.000 (5)	0.007 (5)	0.010 (6)
C18	0.046 (7)	0.043 (7)	0.054 (7)	−0.005 (6)	0.007 (5)	0.005 (6)
C19	0.044 (7)	0.051 (7)	0.064 (7)	−0.001 (6)	0.012 (5)	−0.001 (6)
C20	0.047 (7)	0.057 (7)	0.071 (7)	0.007 (6)	0.013 (6)	−0.003 (6)
C21	0.043 (7)	0.056 (7)	0.076 (8)	0.006 (6)	0.008 (6)	0.006 (7)
C22	0.038 (6)	0.051 (7)	0.055 (6)	−0.001 (5)	0.000 (5)	0.005 (5)
C23	0.043 (7)	0.043 (6)	0.047 (6)	0.003 (5)	0.000 (5)	−0.004 (5)
C24	0.049 (9)	0.042 (7)	0.047 (7)	−0.006 (6)	0.009 (6)	0.002 (6)
C25	0.069 (9)	0.056 (8)	0.064 (7)	0.005 (7)	0.017 (6)	−0.003 (6)
C26	0.090 (11)	0.049 (7)	0.063 (7)	−0.002 (7)	0.001 (7)	−0.009 (6)
C27	0.082 (12)	0.066 (10)	0.060 (9)	0.019 (8)	0.008 (8)	−0.011 (8)
C28	0.064 (8)	0.079 (10)	0.064 (8)	0.010 (7)	0.020 (6)	−0.002 (7)
C29	0.054 (8)	0.065 (8)	0.059 (7)	−0.001 (6)	0.012 (6)	−0.002 (6)
C30	0.052 (7)	0.040 (6)	0.054 (6)	0.002 (5)	0.008 (5)	0.003 (5)
C31	0.075 (10)	0.062 (9)	0.066 (8)	0.010 (8)	0.002 (7)	0.019 (7)
C32	0.088 (11)	0.067 (9)	0.077 (9)	0.003 (8)	0.010 (7)	0.028 (7)
C33	0.093 (11)	0.052 (8)	0.081 (9)	0.002 (8)	0.019 (8)	0.013 (7)
C34	0.089 (11)	0.049 (8)	0.079 (9)	−0.006 (7)	0.015 (8)	0.003 (7)
C35	0.084 (9)	0.050 (7)	0.063 (8)	−0.005 (7)	−0.022 (6)	0.009 (6)
C36	0.053 (9)	0.055 (9)	0.055 (9)	0.014 (7)	0.010 (7)	0.004 (7)
C37	0.072 (9)	0.078 (10)	0.062 (8)	−0.002 (7)	0.006 (7)	0.014 (7)
C38	0.086 (11)	0.092 (12)	0.059 (8)	0.011 (10)	0.008 (7)	0.018 (8)
C39	0.091 (12)	0.069 (11)	0.066 (9)	0.021 (9)	0.029 (9)	0.025 (8)
C40	0.100 (13)	0.058 (9)	0.082 (10)	0.008 (8)	0.033 (9)	0.008 (8)
C41	0.084 (10)	0.063 (9)	0.053 (7)	−0.001 (7)	0.016 (6)	0.008 (6)
C42	0.073 (10)	0.063 (9)	0.077 (8)	−0.001 (7)	0.002 (7)	0.005 (7)
C43	0.100 (12)	0.071 (10)	0.098 (11)	−0.005 (9)	0.002 (9)	0.004 (8)
C44	0.068 (9)	0.075 (9)	0.076 (8)	0.009 (7)	0.010 (7)	−0.004 (7)
C45	0.109 (14)	0.098 (13)	0.107 (12)	0.022 (11)	0.026 (12)	−0.022 (12)
C46	0.067 (9)	0.062 (9)	0.085 (9)	0.006 (7)	−0.003 (7)	−0.012 (8)
C47	0.092 (12)	0.092 (12)	0.078 (10)	0.017 (9)	−0.024 (8)	−0.001 (9)

### Geometric parameters (Å, °)

**Table:**

Sn1—C36	2.154 (15)	C23—H23A	0.9700
Sn1—C24	2.157 (14)	C23—H23B	0.9700
Sn1—C30	2.180 (11)	C24—C25	1.403 (19)
Sn1—O4	2.227 (12)	C24—C29	1.412 (19)
Sn1—O1	2.314 (12)	C25—C26	1.410 (17)
N1—C44	1.507 (16)	C25—H25	0.9300
N1—C46	1.507 (16)	C26—C27	1.37 (2)
N1—C42	1.531 (16)	C26—H26	0.9300
N1—H1	0.9100	C27—C28	1.39 (2)
O1—C1	1.307 (15)	C27—H27	0.9300
O2—C1	1.248 (13)	C28—C29	1.398 (17)
O3—C4	1.371 (17)	C28—H28	0.9300
O3—H3	0.8200	C29—H29	0.9300
O4—C12	1.263 (15)	C30—C35	1.344 (17)
O5—C12	1.276 (14)	C30—C31	1.429 (17)
O6—C15	1.358 (12)	C31—C32	1.387 (19)
O6—H6	0.8200	C31—H31	0.9300
C1—C3	1.502 (15)	C32—C33	1.41 (2)
C2—C3	1.390 (15)	C32—H32	0.9300
C2—C7	1.413 (15)	C33—C34	1.37 (2)
C2—H2	0.9300	C33—H33	0.9300
C3—C4	1.44 (2)	C34—C35	1.382 (18)
C4—C5	1.40 (2)	C34—H34	0.9300
C5—C6	1.456 (16)	C35—H35	0.9300
C5—C23	1.509 (16)	C36—C41	1.38 (2)
C6—C7	1.406 (15)	C36—C37	1.401 (19)
C6—C11	1.431 (15)	C37—C38	1.398 (18)
C7—C8	1.442 (16)	C37—H37	0.9300
C8—C9	1.363 (16)	C38—C39	1.39 (2)
C8—H8	0.9300	C38—H38	0.9300
C9—C10	1.386 (17)	C39—C40	1.37 (2)
C9—H9	0.9300	C39—H39	0.9300
C10—C11	1.340 (17)	C40—C41	1.399 (18)
C10—H10	0.9300	C40—H40	0.9300
C11—H11	0.9300	C41—H41	0.9300
C12—C14	1.505 (16)	C42—C43	1.537 (19)
C13—C14	1.379 (19)	C42—H42A	0.9700
C13—C18	1.39 (2)	C42—H42B	0.9700
C13—H13	0.9300	C43—H43A	0.9600
C14—C15	1.420 (14)	C43—H43B	0.9600
C15—C16	1.400 (15)	C43—H43C	0.9600
C16—C17	1.453 (15)	C44—C45	1.49 (2)
C16—C23^i^	1.520 (15)	C44—H44A	0.9700
C17—C22	1.397 (16)	C44—H44B	0.9700
C17—C18	1.450 (17)	C45—H45A	0.9600
C18—C19	1.408 (17)	C45—H45B	0.9600
C19—C20	1.364 (16)	C45—H45C	0.9600
C19—H19	0.9300	C46—C47	1.48 (2)
C20—C21	1.375 (16)	C46—H46A	0.9700
C20—H20	0.9300	C46—H46B	0.9700
C21—C22	1.379 (16)	C47—H47A	0.9600
C21—H21	0.9300	C47—H47B	0.9600
C22—H22	0.9300	C47—H47C	0.9600
C23—C16^ii^	1.520 (15)		
			
C36—Sn1—C24	110.7 (4)	H23A—C23—H23B	106.9
C36—Sn1—C30	139.3 (5)	C25—C24—C29	117.8 (13)
C24—Sn1—C30	109.5 (5)	C25—C24—Sn1	123.5 (10)
C36—Sn1—O4	91.1 (5)	C29—C24—Sn1	118.3 (10)
C24—Sn1—O4	89.3 (5)	C24—C25—C26	120.3 (13)
C30—Sn1—O4	95.1 (4)	C24—C25—H25	119.8
C36—Sn1—O1	86.3 (5)	C26—C25—H25	119.8
C24—Sn1—O1	89.2 (5)	C27—C26—C25	120.6 (14)
C30—Sn1—O1	88.6 (4)	C27—C26—H26	119.7
O4—Sn1—O1	176.3 (4)	C25—C26—H26	119.7
C44—N1—C46	113.6 (10)	C26—C27—C28	120.5 (14)
C44—N1—C42	114.0 (10)	C26—C27—H27	119.7
C46—N1—C42	109.4 (10)	C28—C27—H27	119.7
C44—N1—H1	106.4	C27—C28—C29	119.7 (13)
C46—N1—H1	106.4	C27—C28—H28	120.2
C42—N1—H1	106.4	C29—C28—H28	120.2
C1—O1—Sn1	114.2 (8)	C28—C29—C24	121.0 (13)
C4—O3—H3	109.5	C28—C29—H29	119.5
C12—O4—Sn1	120.9 (9)	C24—C29—H29	119.5
C15—O6—H6	109.5	C35—C30—C31	117.8 (11)
O2—C1—O1	122.6 (11)	C35—C30—Sn1	123.9 (9)
O2—C1—C3	121.3 (10)	C31—C30—Sn1	118.3 (9)
O1—C1—C3	116.0 (10)	C32—C31—C30	118.1 (15)
C3—C2—C7	121.4 (10)	C32—C31—H31	121.0
C3—C2—H2	119.3	C30—C31—H31	121.0
C7—C2—H2	119.3	C31—C32—C33	122.3 (14)
C2—C3—C4	118.1 (11)	C31—C32—H32	118.9
C2—C3—C1	118.5 (10)	C33—C32—H32	118.9
C4—C3—C1	123.3 (11)	C34—C33—C32	117.6 (13)
O3—C4—C5	118.3 (14)	C34—C33—H33	121.2
O3—C4—C3	119.1 (15)	C32—C33—H33	121.2
C5—C4—C3	122.6 (13)	C33—C34—C35	119.6 (14)
C4—C5—C6	117.7 (12)	C33—C34—H34	120.2
C4—C5—C23	120.1 (11)	C35—C34—H34	120.2
C6—C5—C23	122.1 (12)	C30—C35—C34	124.0 (13)
C7—C6—C11	117.1 (10)	C30—C35—H35	118.0
C7—C6—C5	119.9 (10)	C34—C35—H35	118.0
C11—C6—C5	123.0 (10)	C41—C36—C37	117.0 (14)
C6—C7—C2	120.3 (9)	C41—C36—Sn1	118.1 (11)
C6—C7—C8	119.8 (10)	C37—C36—Sn1	124.9 (12)
C2—C7—C8	119.8 (10)	C38—C37—C36	121.3 (15)
C9—C8—C7	120.0 (11)	C38—C37—H37	119.4
C9—C8—H8	120.0	C36—C37—H37	119.4
C7—C8—H8	120.0	C39—C38—C37	119.4 (14)
C8—C9—C10	119.3 (11)	C39—C38—H38	120.3
C8—C9—H9	120.3	C37—C38—H38	120.3
C10—C9—H9	120.3	C40—C39—C38	120.7 (14)
C11—C10—C9	122.5 (12)	C40—C39—H39	119.6
C11—C10—H10	118.8	C38—C39—H39	119.6
C9—C10—H10	118.8	C39—C40—C41	118.9 (15)
C10—C11—C6	121.0 (11)	C39—C40—H40	120.5
C10—C11—H11	119.5	C41—C40—H40	120.5
C6—C11—H11	119.5	C36—C41—C40	122.5 (14)
O4—C12—O5	123.6 (11)	C36—C41—H41	118.8
O4—C12—C14	118.4 (11)	C40—C41—H41	118.8
O5—C12—C14	118.0 (10)	N1—C42—C43	113.0 (11)
C14—C13—C18	124.6 (15)	N1—C42—H42A	109.0
C14—C13—H13	117.7	C43—C42—H42A	109.0
C18—C13—H13	117.7	N1—C42—H42B	109.0
C13—C14—C15	117.7 (12)	C43—C42—H42B	109.0
C13—C14—C12	121.1 (12)	H42A—C42—H42B	107.8
C15—C14—C12	121.1 (10)	C42—C43—H43A	109.5
O6—C15—C16	118.2 (9)	C42—C43—H43B	109.5
O6—C15—C14	120.0 (9)	H43A—C43—H43B	109.5
C16—C15—C14	121.8 (9)	C42—C43—H43C	109.5
C15—C16—C17	119.2 (10)	H43A—C43—H43C	109.5
C15—C16—C23^i^	119.2 (9)	H43B—C43—H43C	109.5
C17—C16—C23^i^	121.5 (10)	C45—C44—N1	114.9 (12)
C22—C17—C18	117.8 (10)	C45—C44—H44A	108.5
C22—C17—C16	123.4 (11)	N1—C44—H44A	108.5
C18—C17—C16	118.7 (11)	C45—C44—H44B	108.5
C13—C18—C19	124.0 (13)	N1—C44—H44B	108.5
C13—C18—C17	117.8 (13)	H44A—C44—H44B	107.5
C19—C18—C17	118.2 (11)	C44—C45—H45A	109.5
C20—C19—C18	122.3 (11)	C44—C45—H45B	109.5
C20—C19—H19	118.9	H45A—C45—H45B	109.5
C18—C19—H19	118.9	C44—C45—H45C	109.5
C19—C20—C21	118.9 (11)	H45A—C45—H45C	109.5
C19—C20—H20	120.6	H45B—C45—H45C	109.5
C21—C20—H20	120.6	C47—C46—N1	114.0 (12)
C20—C21—C22	122.1 (11)	C47—C46—H46A	108.8
C20—C21—H21	119.0	N1—C46—H46A	108.8
C22—C21—H21	119.0	C47—C46—H46B	108.8
C21—C22—C17	120.8 (11)	N1—C46—H46B	108.8
C21—C22—H22	119.6	H46A—C46—H46B	107.6
C17—C22—H22	119.6	C46—C47—H47A	109.5
C5—C23—C16^ii^	120.3 (11)	C46—C47—H47B	109.5
C5—C23—H23A	107.3	H47A—C47—H47B	109.5
C16^ii^—C23—H23A	107.3	C46—C47—H47C	109.5
C5—C23—H23B	107.3	H47A—C47—H47C	109.5
C16^ii^—C23—H23B	107.3	H47B—C47—H47C	109.5

Symmetry codes: (i) *x*+1, −*y*+1, *z*+1/2; (ii) *x*−1, −*y*+1, *z*−1/2.
